# Learning from Seed Microbes: *Trichoderma* Coating Intervenes in Rhizosphere Microbiome Assembly

**DOI:** 10.1128/spectrum.03097-22

**Published:** 2023-05-17

**Authors:** Penghao Xie, Shengdie Yang, Xiaoyu Liu, Tianyi Zhang, Xinyuan Zhao, Tao Wen, Jian Zhang, Chao Xue, Qirong Shen, Jun Yuan

**Affiliations:** a Jiangsu Provincial Key Lab for Organic Solid Waste Utilization, The Key Laboratory of Plant Immunity, Jiangsu Collaborative Innovation Center for Solid Organic Wastes, Educational Ministry Engineering Center of Resource-Saving Fertilizers, Nanjing Agricultural University, Nanjing, China; b The Key Laboratory of Green Intelligent Fertilizer Innovation, Ministry of Agriculture and Rural Affairs, Nanjing, Jiangsu, China; North-West University

**Keywords:** *T*. *guizhouense* NJAU4742, seed coating, rhizosphere soil microbiome, co-occurrence network, *Trichoderma harzianum*

## Abstract

Seed-associated microbiomes can impact the later colonization of a plant rhizosphere microbiome. However, there remains little insight into the underlying mechanisms concerning how alterations in the composition of the seed microbiome may intervene in the assembly of a rhizosphere microbiome. In this study, the fungus Trichoderma guizhouense NJAU4742 was introduced to both maize and watermelon seed microbiomes by seed coating. Application was found to significantly promote seed germination and improve plant growth and rhizosphere soil quality. The activities of acid phosphatase, cellulase, peroxidase, sucrase, and α-glucosidase increased significantly in two crops. The introduction of *Trichoderma guizhouense* NJAU4742 also led to a decrease in the occurrence of disease. Coating with *T*. *guizhouense* NJAU4742 did not alter the alpha diversities of the bacterial and fungal communities but formed a key network module that contained both *Trichoderma* and *Mortierella*. This key network module comprised of these potentially beneficial microorganisms was positively linked with the belowground biomass and activities of rhizosphere soil enzymes but negatively correlated with disease incidence. Overall, this study provides insights into plant growth promotion and plant health maintenance via seed coating in order to influence the rhizosphere microbiome.

**IMPORTANCE** Seed-associated microbiomes can impact the rhizosphere microbiome assembly and function display. However, there remains little insight into the underlying mechanisms concerning how alterations in the composition of the seed microbiome with the beneficial microbes may intervene in the assembly of a rhizosphere microbiome. Here, we introduced *T*. *guizhouense* NJAU4742 to the seed microbiome by seed coating. This introduction led to a decrease in the occurrence of disease and an increase in plant growth; furthermore, it formed a key network module that contained both *Trichoderma* and *Mortierella*. Our study provides insights into plant growth promotion and plant health maintenance via seed coating in order to influence the rhizosphere microbiome.

## INTRODUCTION

Soil microbes influence important ecosystem-level processes by contributing to nutrient cycling and energy flow (1). Plants host distinct microbial communities that reside within the rhizosphere, phyllosphere, and endosphere, collectively referred to as the “secondary plant genome” (2). The composition and/or function of the plant microbiome has been considered to be a key determinant of plant health and growth (3). Of these plant compartments, the rhizosphere microbiome, which is heavily influenced by root exudates, provides various ecosystem services, such as plant growth promotion and the prevention soilborne pathogen invasion (4). There is ample evidence that soil microbes are recruited by phytochemicals to directly benefit plant growth via the production of phytohormones (such as auxins, cytokinins, and indole-3-acetic acid [IAA]) (5). Soil microbes also display a significant function in activating the activity of some soil enzymes (cellulase and phosphatase) that are involved in organic residue decomposition and nutrient cycling, which accelerates the release of nutrients in soil required for indirectly better plant growth and high yield (6). Furthermore, beneficial microorganisms that inhabit the rhizosphere are known to help plants cope with the environment through various strategies (7). Thus, the utilization of beneficial microbes to promote plant productivity and modulate stress responses is a promising alternative to traditional chemical-based approaches, especially under climate change scenarios. Currently, the application of beneficial microbes to manipulate the plant microbiome is becoming more recognized as an approach that can increase the sustainability of agricultural production systems. However, a critical issue is the continued occupation of these beneficial microbes within rhizosphere soil over time. Several functionally oriented microbes have been introduced to the rhizosphere of crops, such as rhizobia, *Azospirillum*, mycorrhizal fungi, and other biocontrol agents (8, 9). Microbes are typically introduced to a plant rhizosphere through root irrigation or are applied in concert with other substrates, such as organic fertilizer. However, these strategies may be compromised due to poor colonization of the inoculants within the rhizosphere (10).

The interior of seeds harbors a diverse microbial community that establishes mutualistic and pathogenic relationships with plants (11). Studies showed that the majority of pathogenic fungi carried within seeds belong to the genus Fusarium (12, 13), such as Fusarium oxysporum, which causes disease in watermelon and cucumber; and the complex species of Fusarium verticillioides and Fusarium graminearum, which cause stalk rot and ear rot in maize. It has been reported that 25% of global food crops are affected by seed-borne microbes yearly, greatly endangering agricultural production and human health. Seed-borne microbes can influence host plants by altering the assembly of the microbiome (14). This phenomenon may offer a potential strategy on how to “play” the rhizosphere microbiome from seed origins. Plants can vertically transmit beneficial microbes from their ancestors to the next generation via seeds (15). It has also been demonstrated that pathogens can also be carried vertically, resulting in negative impacts on plant descendants (16). Therefore, engineering a rhizosphere microbiome through seed inoculation of beneficial microorganisms has the capability to not only mitigate negative impacts of pathogens but also increase plant growth and stress tolerance. Seed coating with microbial inoculants (e.g., plant growth-promoting bacteria, rhizobia, and arbuscular mycorrhizal fungi [AMF]), plant growth regulators, and micronutrients with the help of a binder has been shown to promote crop productivity (17, 18). This coating would be expected to establish an initial, engineered association between the seed and the soil microbiome by influencing the seed microenvironment. Developing a better understanding of the interactions between seed-borne microbes and, over time, host offspring microbes has important implications for engineering more complex microbial communities in order to produce the intended beneficial effects in a sustainable fashion. However, it remains unknown how the vertical transmission of beneficial microbes by seed coating affects the plant rhizosphere microbiome as well as the specific associated benefits to plant performance.

Members of a fungus, *Trichoderma*, have been widely used as biocontrol agents against plant pathogens through the production of antifungal substances and nutrient competition through the ability to occupy a nutritional niche (19). It has been reported that the application of *Trichoderma* sp. could effectively control Fusarium wilt disease arising from Fusarium oxysporum infections of banana, tomato, and melon plants (20). Furthermore, *Trichoderma* sp. has also exhibited the potential to promote plant growth by increasing nutrient uptake and photosynthetic capacity (21). Currently, many studies are reporting that coating beneficial microorganisms can improve plant health (22, 23). However, previous studies missed the effect of the coated microbiome on the host rhizosphere microbial community. In this study, we coated seeds of maize as well as seeds of watermelon with Trichoderma guizhouense NJAU4742 to evaluate its plant-growth-promoting effect and to assess its influence on the rhizosphere microbial community assembly. We hypothesized that (i) seeds coated with *T*. *guizhouense* NJAU4742 would improve agronomic traits of crops and (ii) the impact of coating *T*. *guizhouense* NJAU4742 on crop performance may be partially explained by a shift in the rhizosphere microbial community.

## RESULTS AND DISCUSSION

### Seed coating with *Trichoderma* sp. improves plant growth and rhizosphere soil enzyme activities.

**FIG 1:**
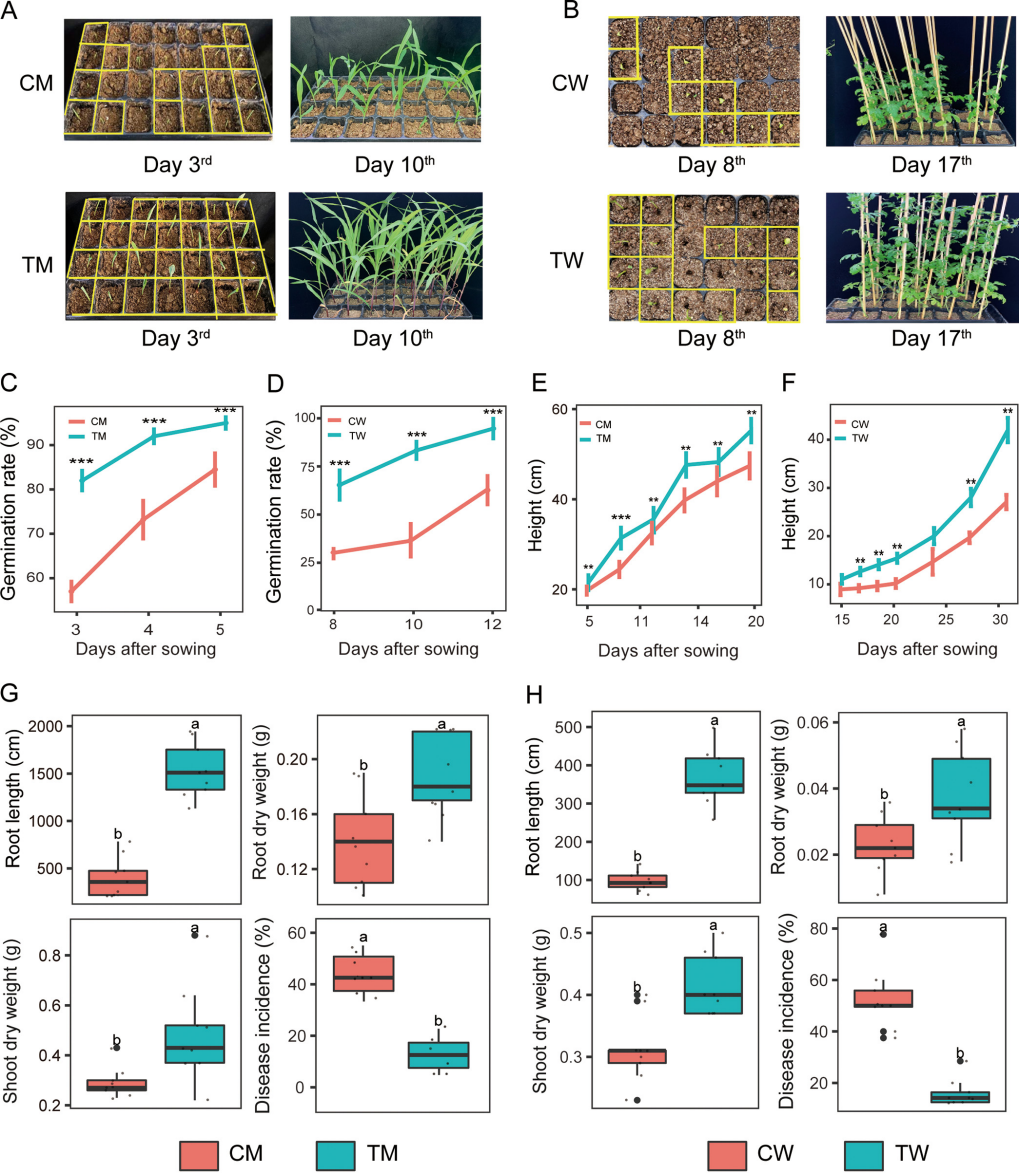
Effect of seed coating with *T. guizhouense* NJAU4742 on plant growth and disease incidence. (A and B) Photographs show the plant-growth-promoting effects on maize and watermelon after sowing (A, maize; B, watermelon). (C and D) The germination rate of maize and watermelon after seeds were coated with or without *T*. *guizhouense* NJAU4742. (E and F) The plant height of maize and watermelon after seeds were coated with or without *T*. *guizhouense* NJAU4742. (G) Plant root height, root dry weight, shoot dry weight, and disease incidence of maize after seeds were coated with or without *T*. *guizhouense* NJAU4742. (H) Plant root height, root dry weight, shoot dry weight, and Fusarium wilt incidence of watermelon after seeds were coated with or without *T*. *guizhouense* NJAU4742. Error bars indicate the standard error based on six replicates. **, 0.001 < *P < *0.01; ***, *P < *0.001. TM, maize seeds coated with *T*. *guizhouense* NJAU4742; CM, maize seeds coated without *T*. *guizhouense* NJAU4742; TW, watermelon seeds coated with *T*. *guizhouense* NJAU4742; CW, watermelon seeds coated without *T*. *guizhouense* NJAU4742.

At the early stage of a plant’s life span, seedlings are exposed to various biotic and abiotic stresses, such as pathogens, herbivores, and drought (24). Thus, manipulating the plant rhizosphere microbiome at an early stage via seed coating with *T*. *guizhouense* NJAU4742 would be expected to promote plant growth and reduce disease incidence. Here, we tested the promotional effect of *T*. *guizhouense* NJAU4742 coating on watermelon and maize. Results showed that the germination rate of coated seeds were significantly increased by 25% for maize after 3 days postplanting (Fig. 1A and C) and 35% for watermelon after 8 days postplanting, compared with the control (Fig. 1B and D). Seed coating was also found to benefit plant growth, as plant height, stem diameter, and biomass production were significantly higher than in the control groups (see Table S2 in the supplemental material). Compared with the control, the final plant height of maize and watermelon was also improved by 20% and 50% (Fig. 1E and F) with a 24% increase in dry shoot weight for watermelon as well as a 39% and 57% increase in dry shoot weight and stem diameter for maize, respectively (Fig. 1G and H; Table S2). Since the plant root system plays a vital role in nutrient uptake and physical support, increasing root biomass and improving root architecture are some of the principal goals of plant breeding (25). Here, seed-coated maize and watermelon exhibited a higher number of primary and lateral roots with the main root lengths significantly elongated by 58% and 72%, respectively, than the control plants (Fig. 1G and H; see Fig. S1 in the supplemental material). This finding was accompanied by an increase of 22% (maize) and 39% (watermelon) in dry root weight (Fig. 1G and H).

Earlier studies have indicated that *Trichoderma* sp. activates soil enzymes and promotes plant growth (26). To address this information, we explored the improvement in enzyme activities within rhizosphere soil after seed coating. Compared with the control, the enzyme activities of urease, sucrase, acid phosphatase, alkaline phosphatase, α-glucosidase, peroxidase, and cellulase increased by 97.9%, 51.7%, 38.3%, 51.6%, 61.2%, 82.3%, and 27.0%, respectively, in the coated maize (see Fig. S2 and Table S3 in the supplemental material). The enzyme activities of sucrase, acid phosphatase, neutral phosphatase, α-glucosidase, peroxidase, polyphenol oxidase, and cellulase increased by 51.7%, 25.7%, 21.1%, 62.6%, 39.1%, 34.3%, and 32.8%, respectively, in the coated watermelon (Fig. S2; Table S3). Cellulase, peroxidase, sucrase, acid phosphatase, and α-glucosidase have been reported to perform vital roles in the hydrolysis of organic matter that releases essential plant nutrients, β-glucosidase, phosphatase, and urease that are useful biological soil quality indicators (27).

Previous research has shown that the inoculation of *Trichoderma* sp. can promote plant growth and reduce the disease occurrence of Fusarium wilt (28). To this end, we explored whether the method of coating with *T*. *guizhouense* NJAU4742 can achieve the same results. We found that the incidence of maize stalk rot in the coated treatment was significantly reduced by 28% compared with that in the control (Fig. 1G), and the incidence of watermelon with coating was reduced by 37% (Fig. 1H) at 60 days after pathogen application. *Trichoderma* spp. have been used widely as biocontrol agents in commercial crop production with previous methods of application principally using combinations of bioorganic fertilizer and microbial inoculum (29). Beneficial effects depend primarily on the metabolic activity of *Trichoderma* sp. as well as beneficial interactions with host plants (30). For example, this strain of *Trichoderma* was reported to efficiently stimulate lateral roots by the secretion of swollen protein (31). In summary, the seed coating strategy implemented here appears to be a high-throughput, lower cost method to achieve similar results to rhizosphere application.

### Seed coating with *Trichoderma* sp. intervenes in rhizosphere microbiome assembly.

Microbial community composition and diversity are critical for maintaining agroecosystem sustainability and productivity (32). Since it was apparent that seed coating was resulting in beneficial effects on plant growth and health, it was necessary to further explore the effect of seed coating on the rhizosphere microbiome. Several studies have reported the reduction of rhizosphere microbial diversity after application of *Trichoderma* sp. as a biofertilizer, resulting in lower complexity and stability of the rhizosphere microbial community (33, 34). Our results showed that coating with *T*. *guizhouense* NJAU4742 did not affect rhizosphere bacterial and fungal diversity (see Fig. S3A and B in the supplemental material), indicating the “safety” of *T*. *guizhouense* NJAU4742 coating on the rhizosphere microbiome. At the phylum level, the relative abundance of *Proteobacteria* was the highest, followed by *Acidobacteria*, and *Gemmatimonadetes* having a relatively high abundance. Moreover, in coated samples with both maize and watermelon, there was no significant difference in the relative abundance of bacteria compared with the control, except for *Bacteroidetes*, which were higher than in the control (Fig. S3C). Accordingly, the subgroups *Chryseolinea*, *Crocinitomix*, and *Niabella* were significantly enriched in the seed-coating treatment (see Table S4 in the supplemental material). These groups may potentially act antagonistically against plant pathogens, as it has been reported that *Chryseolinea* was found to be the key taxa contributing to the microbiome in disease suppressive soils against banana Fusarium wilt (35). Other studies have reported that growth-promoting bacteria, such as *Pseudoxanthomonas* (36), *Herbaspirillum* (37), and *Flavobacterium* (38), were enriched in the rhizosphere of coated watermelon and maize seeds and that they may promote plant growth by altering root architecture and producing various phytohormones (39). In terms of fungal composition, the dominant phylum present was *Ascomycota* which represented 45% of the relative abundance at the phylum level (Fig. S3C). Previously, we found lower abundances of *Ascomycota* and *Basidiomycota* in non-Fusarium wilt diseased soils (40), indicating that these two phyla were not beneficial for plant health in the context of Fusarium wilt disease. Here, the relative abundances of *Ascomycota* and *Basidiomycota* were lower in the coated treatment (Fig. S3C). As expected, the relative abundance of *Trichoderma* sp. in the coated treatment was increased within both the maize and watermelon rhizosphere fungal communities, indicating the effective rhizosphere colonization of *Trichoderma* sp. via seed coating (see Table S6 in the supplemental material).

**FIG 2:**
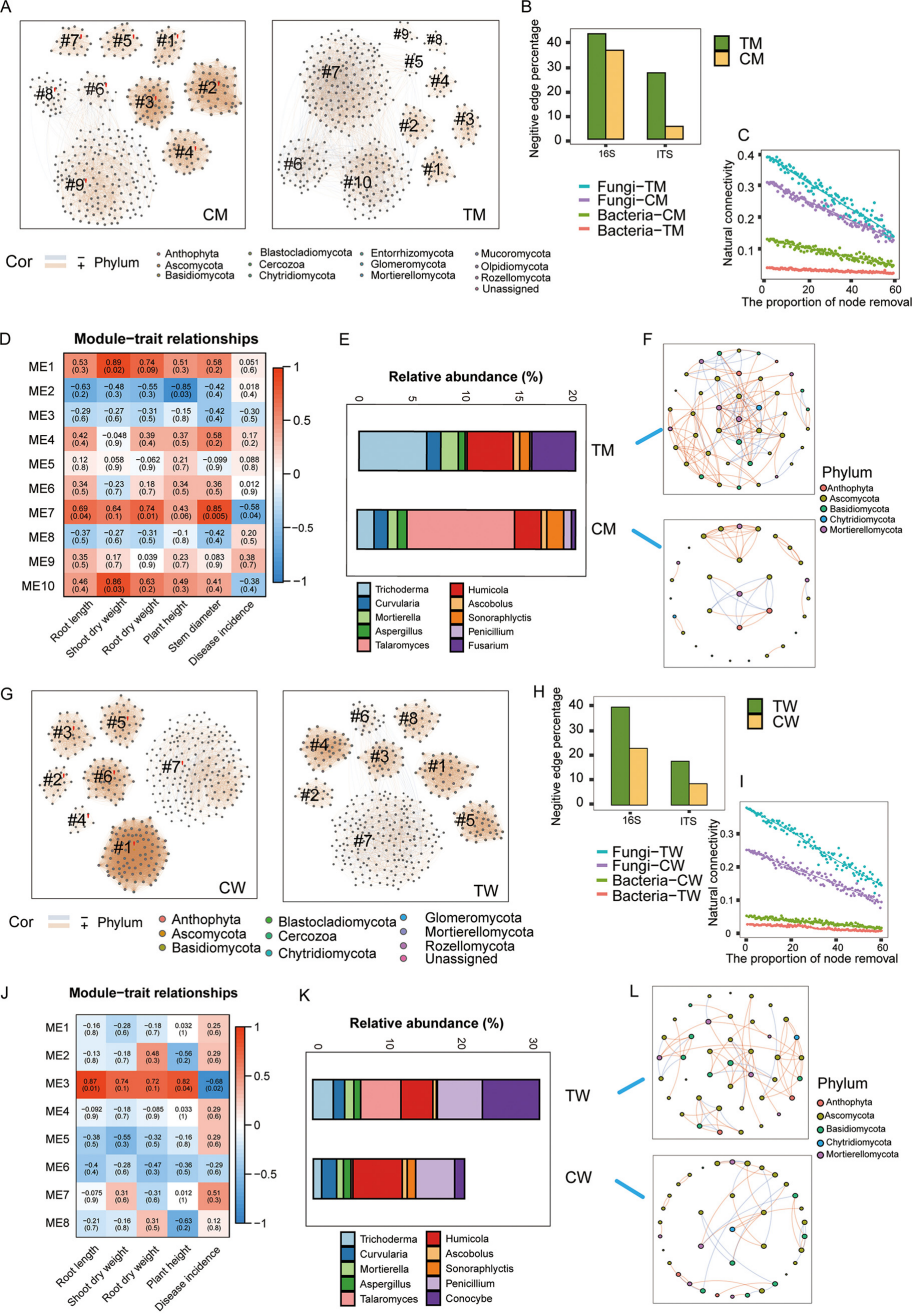
Effect of seed coating with *T. guizhouense* NJAU4742 on rhizosphere microbiome. (A) Co-occurrence network analysis with fungal OTUs of the maize rhizosphere microbiome after seeds were coated with or without *Trichoderma*. Nodes represent OTU, and edges represent significant interactions among nodes (ρ > |0.8|, *P* < 0.05). (B) The ratio of negative edges in the maize rhizosphere microbial network. (C) The stability of microbial networks in the coated and control maize. (D) The correlations between module eigengenes of coated maize fungal network and plant physiological indicators. Red color means positive correlation, and blue color means negative correlation. The numbers in parentheses represent significance (*P*) and the other number without parentheses represents the correlation coefficient (*r*). (E) The relative abundance of OTUs from submodule 7 (TM) in control and coated maize. (F) Co-occurrence networks of OTUs from submodule 7 (TM) in control and coated maize. (G) Co-occurrence network analysis with fungal OTUs of the watermelon rhizosphere microbiome after seeds were coated with or without *Trichoderma*. Nodes represent OTU, and edges represent significant interaction among nodes (ρ > |0.8|, *P* < 0.05, the first value represents correlation and the second value represents significance). (H) The ratio of negative edges in the watermelon rhizosphere microbial network. (I) Display of the stability of the microbial network in the coated and control watermelon. (J) The correlations between module eigengenes and plant physiological indicators in coated maize fungal network. Red color means positive correlation, and blue color means negative correlation. The numbers in each plot are the correlation coefficient (*r*) and significance (*P*) in parentheses. (K) The relative abundance of OTUs from submodule 3 (TW) in control and coated watermelon. (L) The relative abundance of OTUs from submodule 3 (TW) in control and coated watermelon.

In complex ecosystems, co-occurrence networks have been used to explore interactions between microbes. Here, bacterial and fungal co-occurrence networks were constructed based on Spearman correlations among operational taxonomic units (OTUs) to investigate how *T*. *guizhouense* NJAU4742 coating impacted microbial interactions in the rhizosphere soil. Based on network topological comparisons, we found that the proportion of negative edges in both bacterial and fungal networks increased in the seed-coating treatment (Fig. 2B and H; see Table S5 in the supplemental material). This finding indicates that coating with *T*. *guizhouense* NJAU4742 may improve the stability of microbial communities by increasing microbial competition. To further evaluate community stability, natural connectivity was tested by the random removal of nodes. By removing the same proportion (from 1% to 60%) of nodes, results showed that the natural connectivity of fungal networks in coated maize and watermelon was higher than that in the control which indicated that coating with *T*. *guizhouense* NJAU4742 could enhance fungal community stability (Fig. 2C and I). Traditionally, the resilience and stability of beneficial microbial communities in response to a disturbance and their ability to moderate or suppress invasion and disease caused by a pathogen are some of the most important characteristics of ecosystem and organismal health (41). Here, the rhizosphere microbiome stability was shown to be associated with plant health, especially against soilborne diseases.

**FIG 3:**
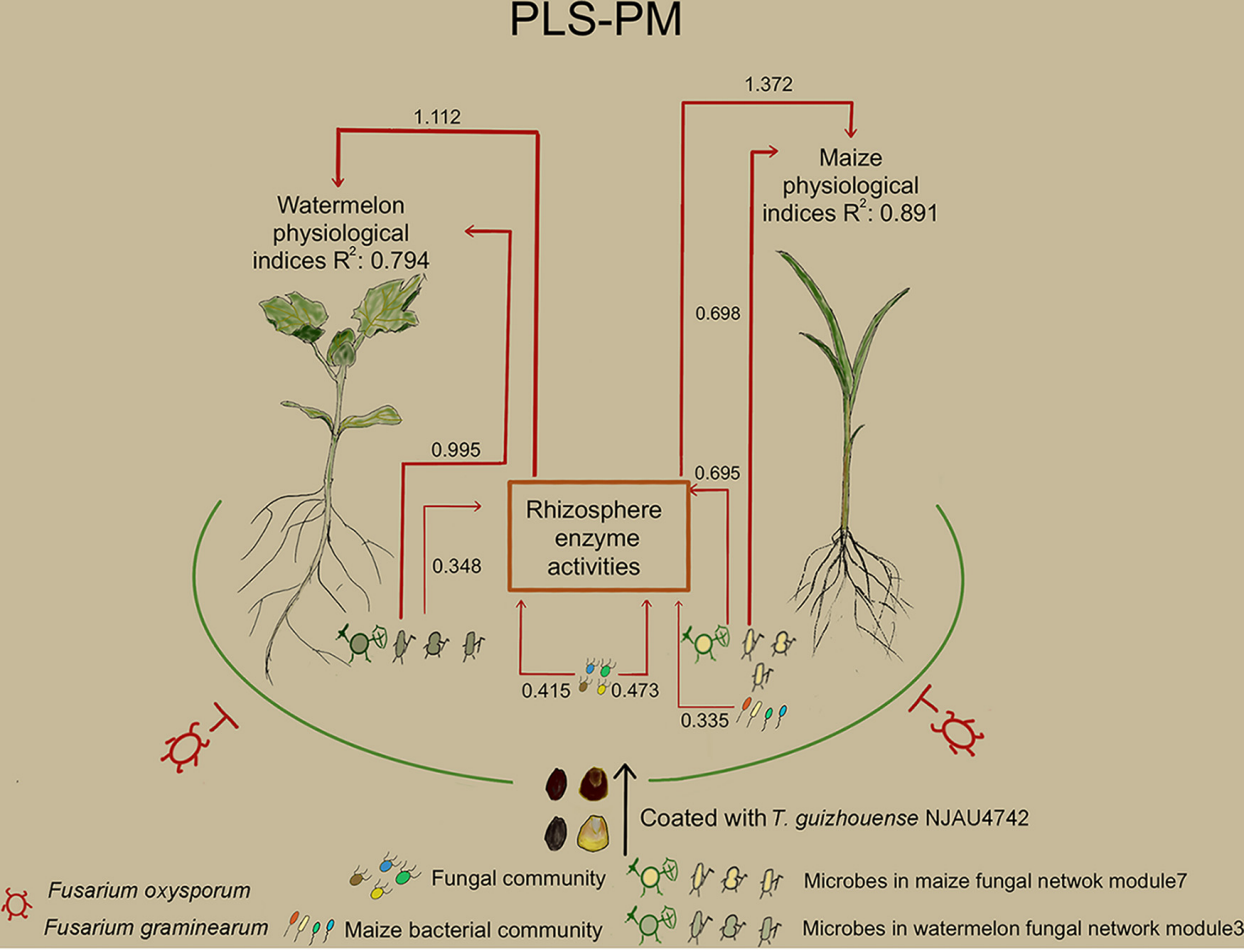
Conceptual diagram with the partial least-squares path model illustrating that coating seeds with *T. guizhouense* NJAU4742 can promote plant growth and help prevent plant disease. PLS-PM shows the direct and indirect pathways through the microbial community that affect plant physiological indices. The red lines indicate positive links. Arrow widths correspond with the relative effect size of each variable. Numbers associated with each arrow are path coefficients (*P* < 0.05). R^2^ indicates variance in plant physiological indices explained by the model.

To evaluate the potential relationship between plant biomass and rhizosphere bacterial/fungal OTUs, we calculated correlations between network module-based eigengenes and plant biomass. In bacterial networks, there was no significant correlation between network modules and plant physiological traits (see Fig. S4 in the supplemental material). For fungal networks, only the coated samples exhibited a significant correlation between network modules and plant physiological traits (Fig. 2D and J). In the fungal network of coated maize, submodule 7 was positively correlated with root length and stem diameter (*P < *0.01) while negatively related to disease incidence (*P < *0.05) (Fig. 2D), and in the fungal network of coated watermelon, submodule 3 was positively correlated with root length and root dry weight (*P < *0.01) while negatively related to disease incidence (*P < *0.05) (Fig. 2J). Moreover, in coated maize samples, the submodule 7 was positively correlated with urease (*r* = 0.75), acid phosphatase (*r* = 0.78), alkaline phosphatase (*r* = 0.76), and cellulase (*r* = 0.8) enzyme activities. In coated watermelon samples, the submodule 3 was positively correlated with α-glucosidase (*r* = 0.78), polyphenol oxidase (*r* = 0.6), and cellulase (*r* = 0.85) enzyme activities (see Fig. S5 in the supplemental material). The partial least-squares path model (PLS-PM) analysis also showed that the microbial communities of fungal submodule 7 (maize) and submodule 3 (watermelon) in the coating treatment might affect plant growth directly or indirectly by improving enzyme activities in the rhizosphere soil (Fig. 3). These results suggested that the enhancement of rhizosphere soil enzyme activities was principally driven by the microorganisms within fungal submodule 7 (maize) and submodule 3 (watermelon). Previous findings have demonstrated clear linkages between rhizosphere fungal communities and rhizosphere enzyme activities (8). One proposed explanation was that soil fungi were the most vital contributor to soil enzymes which improved soil quality, further promoting the increase in plant biomass (42). Further analysis showed a variety of plant mutualists, including *Trichoderma* and *Mortierella* (43, 44), that were enriched in submodule 7 and submodule 3 (Fig. 2E and K; Table S6), and they were identified as key hubs with high betweenness centrality and degree (Fig. 2F and L; see Table S7 in the supplemental material). These essential microorganisms, such as *Trichoderma* sp., possess high potency to produce enzymes (45).

To further validate whether the formation of submodule 3 and submodule 7 was predominantly driven by the introduction of *Trichoderma* sp., we recalculated the microbiome co-occurrence network of maize seed coating treated with spores (TM) and watermelon seed coating treated with spores (TW) after removing the *Trichoderma* genus from the OTU table or reducing the abundance of *Trichoderma* sp. in the coated samples to that of the control. None of the modules in the reconstructed coated fungal networks, after the removal of the *Trichoderma* genus, were found to be similar to the previous submodule 7 (maize) and submodule 3 (watermelon) by comparing the proportion of shared nodes in the two targeted modules (see Table S8 in the supplemental material). Identical results were found after reducing the abundance of *Trichoderma* sp. in the coated maize. One of the modules in the coated watermelon (by reducing the abundance of *Trichoderma*) was similar to the previous submodule 3 (watermelon), but five shared OTUs were not the key hubs in submodule 3 (watermelon) (Table S8). Network hubs have been proposed as keystone taxa and ecological generalists critical for the maintenance of community structure, function, and overall ecosystem stability (46). In this study, we found that seed coating with *Trichoderma* sp. modulated the function and structure of rhizosphere fungal communities and enriched a variety of fungi associated with plant growth and health.

### Conclusion.

Our study evaluated the effect of seeds coated with *T*. *guizhouense* NJAU4742 on plant growth and the associated rhizosphere microbial community. The results revealed that seed coating enhanced seed germination and improved the root architecture of the plants, including increasing the main root length and the number of primary and lateral roots. In addition, it also increased the above/belowground biomass of the plant and reduced the occurrence of disease. Furthermore, some growth-promoting microbes like *Trichoderma* and *Mortierella* in the coated microbiome were found to principally contribute to the positive correlations between the microbiome composition and function (plant growth promotion and disease resistance). Our results demonstrated that the manipulation of the rhizosphere microbiome by seed coating with *T*. *guizhouense* NJAU4742 was an efficient strategy to achieve plant growth promotion as well as effective disease control.

## MATERIALS AND METHODS

### Preparation of *Trichoderma*-containing seed coating agent.

The coating agent used in this experiment was prepared by adding 1% starch and 10% iron oxide red (coloring agent) to a prepared *T*. *guizhouense* NJAU4742 spore solution (1 × 10^8^ spore/mL). The Trichoderma spore solution was prepared as follows: *T*. *guizhouense* NJAU4742 (GenBank accession number LVVK01000017; stored in our laboratory, and the spores of this strain were preserved in 30% glycerin at −80°C to prevent spawn degeneration) was used in this experiment and stored at −80°C as a spore solution, and 1 mL of spores was placed in 30 mL potato dextrose broth (PDB) and incubated for 7 days at 26°C and 180 rpm in a thermostat incubator chamber. Spores were then harvested by filtering three times with sterile gauze to remove mycelium. The spore concentration was adjusted to 1 × 10^8^ spore/mL, based on the measurement of the hemocytometer.

### Pot experiment.

We used watermelon (“Lenong No.3”) and maize (“Zhongnuo 309”) to test the biological effect of seed coating with *T*. *guizhouense* NJAU4742. For seed coating with *T*. *guizhouense* NJAU4742, 2 mL of coating agent was used for 100 maize seeds and 1 mL was used for 100 watermelon seeds, resulting in a concentration of *T*. *guizhouense* NJAU4742 spores at 1 × 10^6^ spore per seed. For the control treatments, identical numbers of maize and watermelon seeds were coated with a coating agent comprised of inactivated spores. The soil for the pot experiment was collected from the Baima Teaching and Research Base of Nanjing Agricultural University (31°36′N latitude and 119°10′E longitude). The physicochemical properties of the soil were presented in Table S1 in the supplemental material. One coated seed was planted in each cell (6.8 cm × 7.5 cm × 10cm, containing 100 g soil) of seeding trays (21 cells per tray) that were placed in a greenhouse with 16 h photoperiod (120 μmol photons m^−2^ · s^−1^) at 23/20°C day/night temperatures. Each crop type was comprised of a coated treatment (10^6^ spores per seed) and control, as follows: maize seed coating treated with spores (TM), control maize seed coating with inactivated spores (CM), watermelon seed coating treated with spores (TW), and control watermelon seed coating with inactivated spores (CW). The 2 treatments of maize seeds contained 168 seedlings divided into 6 replicates with 28 seedlings per replicate. The 2 treatments of watermelon seeds contained 144 seedlings divided into 6 replicates with 24 seedlings per replicate.

The seedling germination rate of maize was recorded on the 3rd day after planting. After planting for 8 days, the watermelon seedling germination rate was recorded. The plant height was measured every 3 days until irrigating pathogen solution at the 35th day after planting. At the same time, six plants from each replicate were harvested to measure stem diameter, root parameter (measured by root scanner, GXY-A; Zhejiang Top Cloud Agri Technology Co., Ltd.), and biomass, and rhizosphere soil was collected for subsequent determination.

To test the biocontrol effect of the seed coating with *T*. *guizhouense* NJAU4742, we followed an inoculation method used in previous publications (47, 48). Briefly, watermelon was irrigated with a conidial suspension (Fusarium oxysporum f. sp. *niveum* was isolated in our lab [49]) of 1 × 10^5^ spores per mL. A pathogen for maize (Fusarium graminearum) was kindly provided by Xiao (Heilongjiang Academy of Agricultural Sciences) (48), and the inoculation method was the same as mentioned above. After 25 days of inoculation with the pathogen, the disease incidence did not increase anymore, and we recorded the number of watermelon and maize with obvious disease symptoms (the watermelon seedlings exhibited stunted growth, with yellowing and wilting of the leaves starting from the base of the plant; the maize seedlings exhibited slow growth and stem discoloration). The whole experimental period lasted 60 days.

### Rhizosphere soil genome DNA extraction and sequencing.

Before DNA extraction, 0.5 g of soil adhered tightly with roots was placed into a 2 mL centrifuge tube containing 1 mL of phosphate-buffered saline solution and several sterilized glass beads, after which the mixture was vortexed at maximum speed for 15 min. The suspension (without root materials) was then transferred to a new 2-mL centrifuge tube and centrifuged for 30 min at 15,000 rpm. The supernatant was discarded, and the precipitate was used for DNA extraction. In total, 20 samples were collected and total DNA was extracted from the precipitate using a FastBeat SoilPure PowerSoil DNA Isolation Kit (BOLAZ) according to the manufacturer’s protocol. The DNA quality and quantity were measured via a 1.2% agarose gel and a NanoDrop 1000 spectrophotometer (Thermo Scientific, USA).

Both the ITS1-2 and V4 regions of 16S rRNA gene libraries were sequenced using the Illumina MiSeq platform at Guangdong Magigene Biotechnology, Co., Ltd., China. The primer pair 515F (GTGYCAGCMGCCGCGGTAA) and 806R (GGACTACNVGGGTWTCTAAT) for bacteria and ITS1F (CTTGGTCATTTAGAGGAAGTAA) and ITS2R (GCTGCGTTCTTCATCGATGC) for fungi were used. The barcoded PCR amplicon library was built separately for bacterial and fungal community analysis. Bacterial and fungal amplicon library preparation and high-throughput sequencing were performed as described previously (40). The DNA marker was provided by Sangon Biotech, Shanghai (B500351) with a range of 100–5000 bp.

For sequencing data analyses, usearch (V. 10.1) and vsearch (V. 0.6.3) were implemented. First, the “vsearch –fastq_mergepairs” script was used for merging paired-end sequences, “vsearch –fastx_filter” script was used to cut primers, “vsearch –derep_fulllength” script was used for finding unique read sequences, and “usearch -unoise3” script was used for cluster OTUs. The “usearch -otutab” script was used to create an OTU table. The “vsearch –sintax” script, Silva tax database, and unite tax database were used for bacterial and fungal OTU annotation.

### Rhizosphere soil enzyme activity determination.

In order to characterize whether the introduction of *T*. *guizhouense* NJAU4742 by coating can improve rhizosphere soil quality and promote plant growth, we selected 14 rhizosphere soil enzymes for measurement. The rhizosphere soil (collections are detailed in the section “Pot experiment”) was used to measure activities of the following enzymes in Nanjing Convinced-test, Co., Ltd., China using methodologies from the indicated references: catalase (50), urease (51), sucrase (50), acid phosphatase (52), alkaline phosphatase (53), neutral phosphatase (54), α-glucosidase (55), β-glucosidase (56), peroxidase (57), polyphenol oxidase (58), nitrite reductase (59), leucine aminopeptidase (60), β-*N*-acetylglucosaminidase (61), and cellulase (62). There were six replicates for each treatment, and every replicate was also comprised of six technical replicates.

### Statistical analysis.

Statistical analysis in the study was performed using the statistical programming language R 4.1 (63). Differences between treatments of plant physiological parameters were analyzed by Wilcoxon signed-rank test. The significance threshold was set as adjusted *P* values of <0.05. The *P* values were corrected by the Benjamini-Hochberg false discovery rate (FDR) procedure for multiple comparisons. Visualization analysis was done by R package “ggplot2” (64).

A normalized number of sequences were randomly extracted from each sample to calculate alpha diversity indices with the R package “vegan” (65). Wilcoxon nonparametric tests were used for the detection of significance for Shannon diversity, Pielou evenness, and the Chao1 indexes using the “EasyStat” package. Before the calculation of beta diversity, relative abundances were used to standardize the OTU profiles. Bray-Curtis similarity matrices were prepared using the “vegan” R packages. Permutational multivariate analysis of variance (PERMANOVA; Adonis, transformed data by Bray-Curtis, permutation = 999) was used to test if the beta diversity differed among treatments. Nonmetric multidimensional scaling (NMDS) plots were generated according to Bray-Curtis similarity matrices created using the R package “ggplot2” (66). R package “edgeR” were used to identify differentially abundant microbes between treatments (67). Network analysis was performed using R package “ggClusterNet” (68). In the eigengene analysis, each network module is represented by principal-component analysis (PCA) of the abundance profile called module eigengene “WGCNA” (69). The R package “WGCNA” was used to calculate the correlations between module-based eigengenes and plant physiological enzyme activities. The partial least-squares path model (PLS-PM) was performed to further infer potential direct and indirect effects of the bacterial community, fungal community, network module, and soil enzyme activities on plant physiological indices. All PLS-PM analyses were conducted using R package “plspm” (70). The module paired was calculated according to Yuan (46).

### Data availability.

Raw sequence data obtained in this study have been deposited in the Genome Sequence Archive in the BIG Data Center, Chinese Academy of Sciences under accession code CRA007067.
