# Supplementary material for: Learning from Seed Microbes: Trichoderma Coating Intervenes in Rhizosphere Microbiome Assembly
Source: Microbiol Spectr. 2023 May 17;11(3):e03097-22. doi: 10.1128/spectrum.03097-22 (PMC10269462; doi:10.1128/spectrum.03097-22)
Supplement: Supplemental file 1 — Supplemental material. Download spectrum.03097-22-s0001.pdf, PDF file, 1.6 MB [file spectrum.03097-22-s0001.pdf]

Supplementary Materials for

Learn from seed microbes: *Trichoderma* coating intervenes in rhizosphere microbiome assembly

Supplementary Figure 1: Scanning of roots with root image analysis system. The washed roots are placed in transparent tray and are scanned and analyzed for measurement of various root parameters.

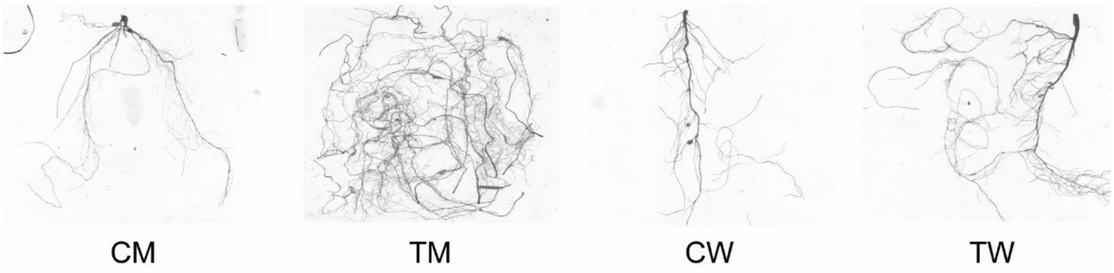

Supplementary Figure 2: Rhizosphere soil enzyme activities of CM, TM, CW, and TW.

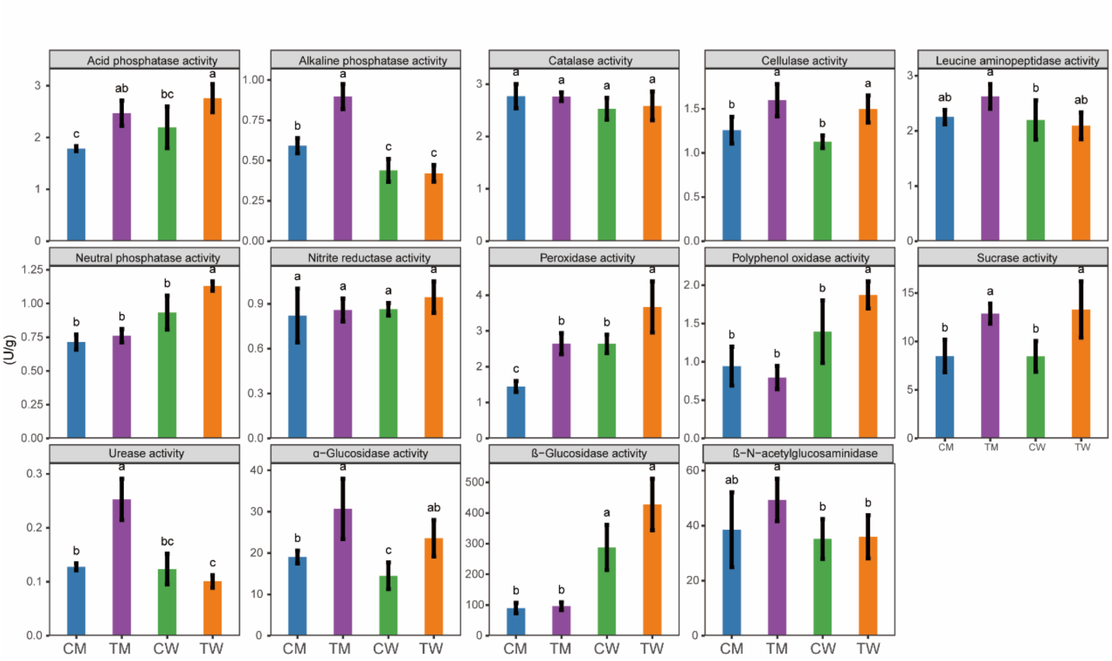

Supplementary Figure 3: Diversity, community structure and composition of bacteria and fungi in rhizosphere soil. A: *Alpha* diversity of the rhizosphere soil bacterial and fungal communities among different treatments. B: Non-metric multidimensional scaling (NMDS) of bacterial and fungal communities. The stress value for bacterial and fungal was 0.14, 0.16. C: Chord plot depicts the relative abundances (%) of major phyla present in the bacterial and fungal communities. The bar graph shows the relative abundance of *Bacteroidetes* in different treatments.

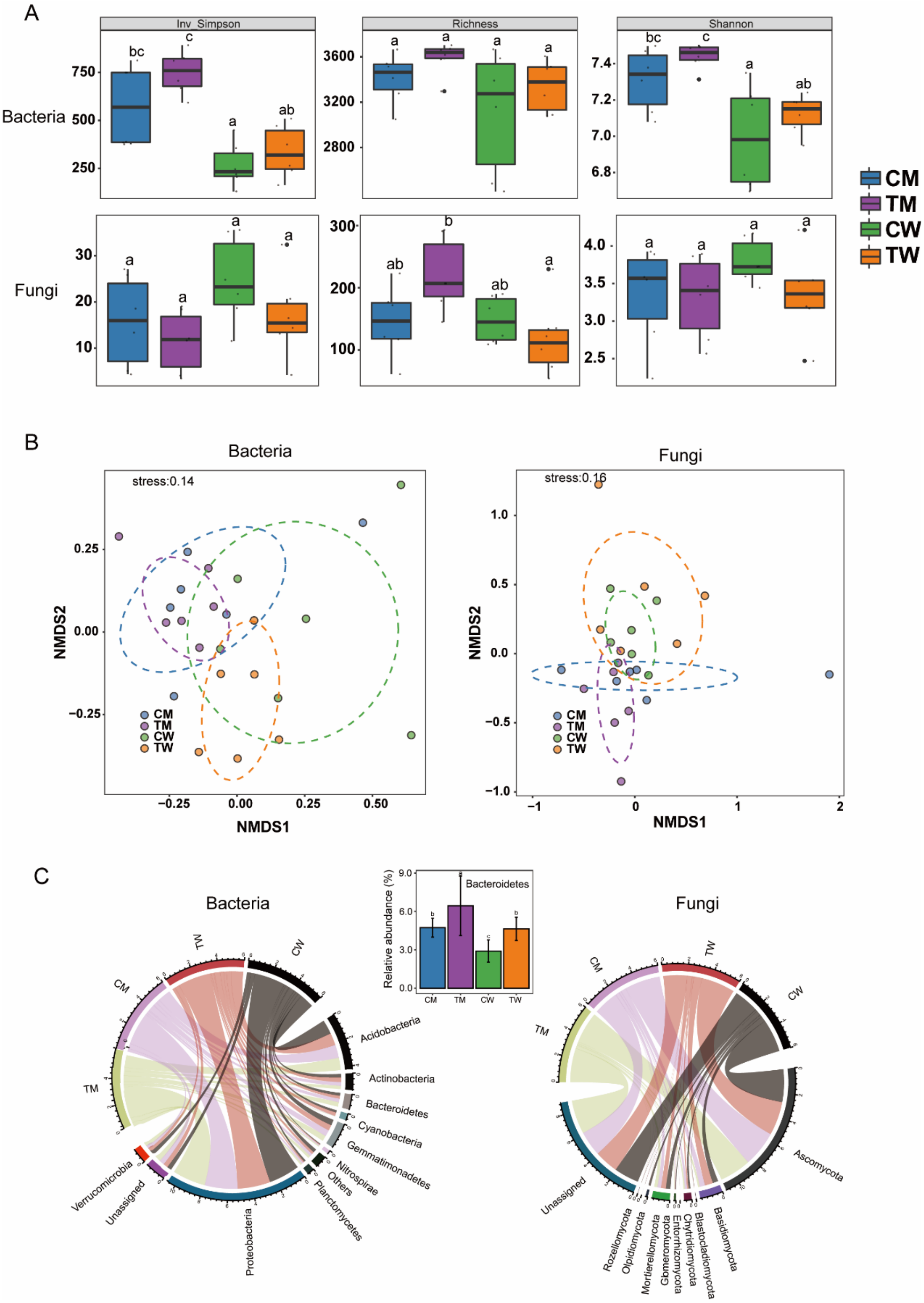

Supplementary Figure 4: The correlations between bacterial module eigengenes and plant physiological indicators.

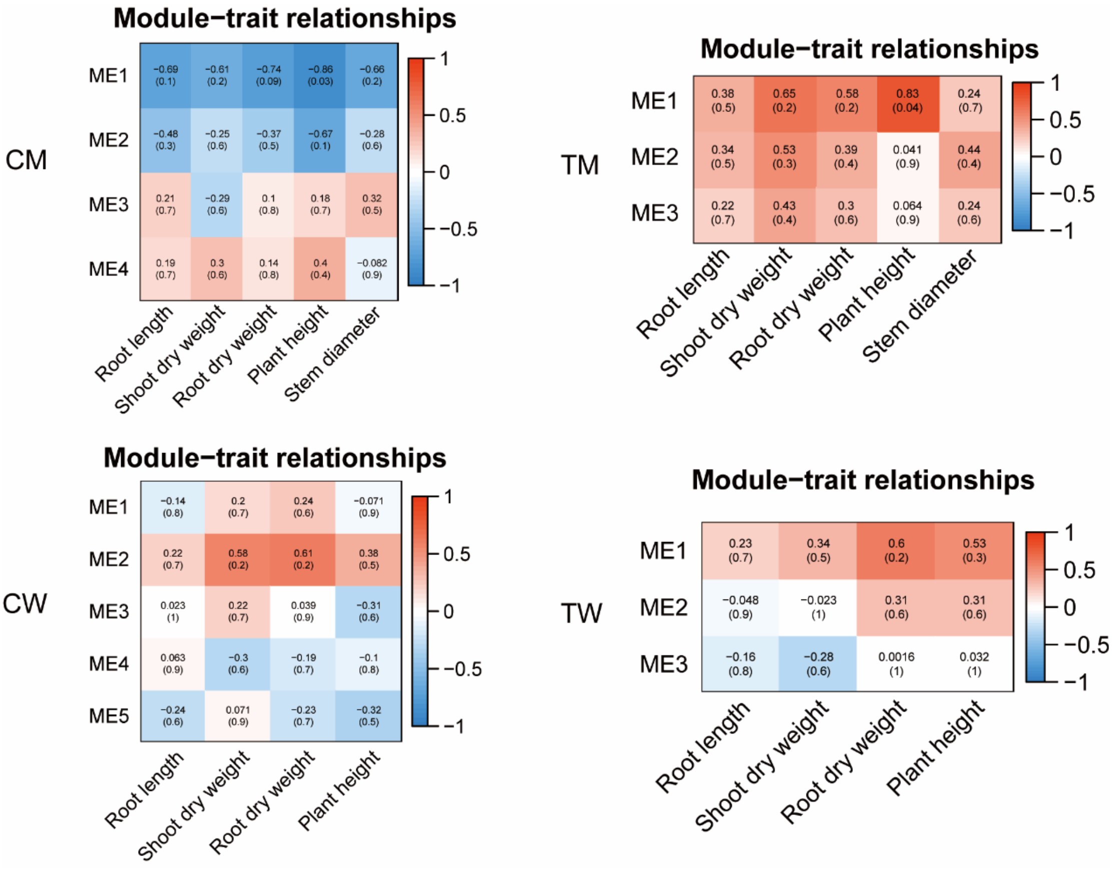

Supplementary Figure 5: The correlations between fungal module eigengenes and rhizosphere soil enzyme activities.

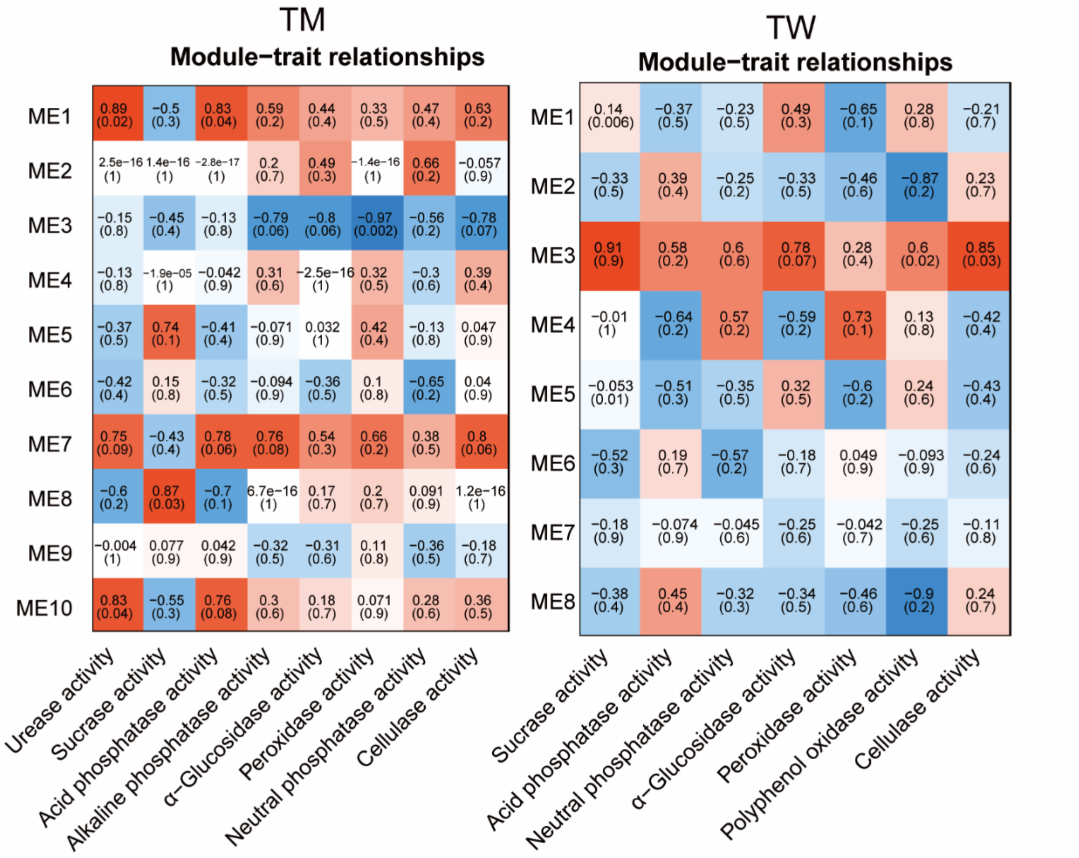

Supplementary Table 1: Physicochemical properties of soil.

AP (mg/kg)	AK (mg/kg)	pH	TK (g/kg)	TN (g/kg)	TC (g/kg)	C/N	TP (g/kg)
41.24± 0.43	45.23± 2.12	7.54± 0.01	1.72± 0.04	1.88± 0.01	15.19± 0.04	8.10± 0.05	0.60± 0.01

Supplementary Table 2: Physiological indices between coated and control treatment.

	Shoot dry weight (g)	Root dry weight (g)	Disease incidence (%)	Root length (%)	Stem diameter (cm)
CW	0.4	0.029	77.7	71.529	NA
CW	0.31	0.019	50	81.6357	NA
CW	0.27	0.036	40	92.4402	NA
CW	0.23	0.033	60	61.529	NA
CW	0.31	0.016	37.5	141.6357	NA
CW	0.31	0.024	50.6	92.4402	NA
TW	0.46	0.02	12.5	328.1097	NA
TW	0.37	0.033	12.5	397.828	NA
TW	0.4	0.034	28.5	257.697	NA
TW	0.47	0.049	14.2	328.1097	NA
TW	0.37	0.018	14.2	497.828	NA
TW	0.5	0.042	12.2	347.697	NA
CM	0.27	0.19	46.1	691.0682	0.1
CM	0.24	0.11	48.0	476.9727	0.2
CM	0.27	0.14	37.6	231.8768	0.1
CM	0.23	0.12	35.5	491.0682	0.2
CM	0.26	0.16	36.2	276.9727	0.1
CM	0.29	0.19	39.5	241.8768	0.1
TM	0.22	0.22	15.8	1500.3113	0.4
TM	0.42	0.18	8.8	1896.2555	0.4
TM	0.51	0.17	20.5	1736.1042	0.4
TM	0.64	0.22	18.4	1132.5988	0.3
TM	0.88	0.22	7.3	1922.4793	0.3
TM	0.52	0.20	11.5	1393.3799	0.3

Note: The abbreviation "NA" stands for "no data available" in this context.

Supplementary Table 3: Rhizosphere enzyme activities of control and coated samples.

	Catalase activity (U/g)	Urease activity(U/g)	Sucrase activity(U/g)	Acid phosphatase activity(U/g)	Alkaline phosphatase activity(U/g)	Neutral phosphatase activity(U/g)	α-glucosidase activity(U/g)	β-glucosidase activity(U/g)
CM	2.728	0.137	7.344	1.759	0.521	0.653	20.014	109.808
CM	2.961	0.122	11.329	1.861	0.652	0.757	20.147	101.016
CM	2.560	0.121	8.647	1.733	0.586	0.651	20.341	94.062
CM	3.051	0.129	7.557	1.786	0.533	0.754	17.324	72.815
CM	2.545	0.127	8.437	1.786	0.600	0.783	16.228	70.386
CM	2.507	0.138	0.684	1.470	0.787	0.999	14.242	70.868
TM	2.861	0.315	11.936	2.450	0.940	0.834	26.578	111.253
TM	2.818	0.211	14.503	2.157	0.925	0.789	20.028	87.068
TM	2.661	0.252	13.016	2.412	0.980	0.734	33.001	105.422
TM	2.696	0.242	12.031	2.488	0.786	0.742	37.245	80.410
TM	2.761	0.244	12.871	2.838	0.852	0.711	36.527	97.995
TM	2.077	0.223	11.001	2.608	0.798	0.645	30.457	95.286
CW	2.305	0.090	8.523	2.380	0.352	0.993	9.344	237.160
CW	2.828	0.098	6.637	2.199	0.407	0.803	13.952	216.281
CW	2.423	0.145	9.232	1.525	0.531	0.734	16.524	305.991
CW	2.645	0.133	7.293	2.293	0.490	0.887	14.979	275.565
CW	2.446	0.152	10.610	2.586	0.413	1.037	17.699	403.593
CW	2.013	0.133	11.634	2.547	0.370	1.113	15.892	350.036
TW	2.781	0.093	10.562	2.853	0.383	1.094	22.117	375.117
TW	2.383	0.109	13.011	3.047	0.427	1.114	29.835	545.432
TW	2.277	0.091	11.553	2.953	0.428	1.167	20.926	488.146
TW	2.937	0.117	18.087	2.507	0.497	1.108	26.185	369.108
TW	2.541	0.093	13.236	2.443	0.365	1.165	18.811	360.750
TW	2.904	0.487	11.298	2.420	0.415	1.241	20.892	450.872

	Peroxidase activity(U/g)	Polyphenol oxidase activity(U/g)	Nitrite reductase activity(U/g)	Leucine aminopeptidase activity(U/g)	β-N-acetylglucosaminidase(U/g)	Cellulase activity(U/g)
CM	1.671	0.680	1.132	2.404	59.054	1.012
CM	1.378	1.297	0.775	2.372	45.893	1.328
CM	1.262	1.108	0.807	2.223	30.275	1.323
CM	1.468	0.815	0.698	2.129	25.871	1.217
CM	1.374	0.860	0.767	2.275	28.667	1.403
CM	1.090	0.877	0.438	2.107	27.045	1.228
TM	2.822	0.612	0.857	2.325	58.077	1.805
TM	2.700	0.779	0.831	2.724	47.835	1.577
TM	2.815	0.715	0.980	2.913	55.675	1.727
TM	2.125	1.014	0.857	2.502	46.089	1.535
TM	2.749	0.855	0.769	2.661	38.951	1.335
TM	2.033	0.936	0.635	2.975	33.942	1.311
CW	2.573	0.733	0.882	1.834	35.731	1.070
CW	2.987	1.367	0.790	1.869	24.630	1.210
CW	2.496	1.598	0.885	2.387	36.866	1.148
CW	2.336	1.827	0.874	2.680	35.517	1.166
CW	2.801	1.446	0.891	2.222	44.805	1.036
CW	2.313	1.597	0.989	2.897	37.972	1.311
TW	3.735	1.842	1.077	1.860	26.159	1.428
TW	3.073	1.592	0.799	2.273	43.663	1.734
TW	4.037	2.065	0.915	2.356	42.134	1.560
TW	2.886	1.949	1.008	2.157	38.614	1.375
TW	4.625	1.913	0.928	1.817	29.085	1.379
TW	4.133	2.792	0.989	2.409	45.635	1.330

Supplementary Table 4: The differential analysis of microbial abundance between control and coated groups.

Group	Differential microbial taxonomy	logFC	Level	P value	MeanR-C	MeanR-T
TM/CM	Pseudoxanthomonas	2.81	Enriched	8E-05	0.00095	0.0067
	Burkholderia	-1.88	Depleted	4E-04	0.01702	0.00459
	Kaistia	2.34	Enriched	2E-03	0.00064	0.003241
	Roseomonas	3.41	Enriched	1E-03	0.00015	0.001592
	Emticicia	2.32	Enriched	1E-03	0.00018	0.000897
	Niabella	3.42	Enriched	7E-05	4.91E-05	0.000527
	Flavobacterium	4.59	Enriched	5E-04	1.82E-05	0.000438
	Crocinitomix	5.59	Enriched	8E-04	3.27E-06	0.000157
	Pantoea	-1.95	Depleted	9E-04	3.89E-05	0.00001
	Umezawaea	-6.95	Depleted	5E-05	0.00010	0.0000008
	Chryseolinea	3.38	Enriched	6E-04	0.00017	0.00177
TW/CW	Actinocorallia	2.84	Enriched	4E-04	0.00011	0.00079
	Crocinitomix	1.74	Enriched	6E-04	0.00003	0.0001
	Niabella	5.05	Enriched	7E-04	6.66E-06	0.00022
	Roseimicrobium	-3.38	Depleted	4E-05	0.00069	6.65E-05
	Chryseolinea	1.65	Enriched	2E-04	0.00027	0.00085
	Herbaspirillum	2.13	Enriched	2E-04	0.000217	0.00095

MeanR-C: relative abundance of bacteria in control

MeanR-T: relative abundance of bacteria in the coated treatment

Supplementary Table 5: Topological properties of the network under fungal and bacterial communities.

	Group	KO	Means	SD
Num.edges	CM-Fungi	5120.000	5120.000	0.000
Num.pos.edges	CM-Fungi	4851.000	0.000	0.000
Num.neg.edges	CM-Fungi	269.000	0.000	0.000
Num.vertices	CM-Fungi	400.000	400.000	0.000
Connectance	CM-Fungi	0.064	0.064	0.000
Average.degree	CM-Fungi	25.600	25.600	0.000
Average.path.length	CM-Fungi	2.935	2.117	0.001
Diameter	CM-Fungi	5.131	3.000	0.000
Edge.connectivity	CM-Fungi	0.000	12.180	1.445
Clustering.coefficient	CM-Fungi	0.936	0.064	0.001
No.clusters	CM-Fungi	7.000	1.000	0.000
Centralization.degree	CM-Fungi	0.054	0.039	0.005
Centralization.betweenness	CM-Fungi	0.011	0.004	0.001
Centralization.closeness	CM-Fungi	0.002	0.082	0.007
Num.edges	TM-Fungi	4295.000	4295.000	0.000
Num.pos.edges	TM-Fungi	3129.000	0.000	0.000
Num.neg.edges	TM-Fungi	1166.000	0.000	0.000
Num.vertices	TM-Fungi	400.000	400.000	0.000
Connectance	TM-Fungi	0.054	0.054	0.000
Average.degree	TM-Fungi	21.475	21.475	0.000
Average.path.length	TM-Fungi	2.823	2.244	0.002
Diameter	TM-Fungi	4.229	3.100	0.302
Edge.connectivity	TM-Fungi	0.000	9.150	1.500
Clustering.coefficient	TM-Fungi	0.631	0.054	0.001
No.clusters	TM-Fungi	7.000	1.000	0.000
Centralization.degree	TM-Fungi	0.044	0.036	0.006
Centralization.betweenness	TM-Fungi	0.008	0.005	0.001
Centralization.closeness	TM-Fungi	0.004	0.094	0.009
Num.edges	CM-Bacteria	3183.000	3183.000	0.000
Num.pos.edges	CM-Bacteria	2024.000	0.000	0.000
Num.neg.edges	CM-Bacteria	1159.000	0.000	0.000
Num.vertices	CM-Bacteria	909.000	909.000	0.000
Connectance	CM-Bacteria	0.008	0.008	0.000
Average.degree	CM-Bacteria	7.003	7.003	0.000
Average.path.length	CM-Bacteria	1.253	3.716	0.006
Diameter	CM-Bacteria	5.914	6.970	0.264
Edge.connectivity	CM-Bacteria	0.000	0.560	0.499
Clustering.coefficient	CM-Bacteria	0.975	0.008	0.001
No.clusters	CM-Bacteria	164.000	1.690	1.051
Centralization.degree	CM-Bacteria	0.017	0.011	0.001

Centralization.betweenness	CM-Bacteria	0.000	0.011	0.002
Centralization.closeness	CM-Bacteria	0.000	0.067	0.022
Num.edges	TM-Bacteria	1710.000	1710.000	0.000
Num.pos.edges	TM-Bacteria	972.000	0.000	0.000
Num.neg.edges	TM-Bacteria	738.000	0.000	0.000
Num.vertices	TM-Bacteria	878.000	878.000	0.000
Connectance	TM-Bacteria	0.004	0.004	0.000
Average.degree	TM-Bacteria	3.895	3.895	0.000
Average.path.length	TM-Bacteria	1.000	5.089	0.031
Diameter	TM-Bacteria	1.000	10.860	0.603
Edge.connectivity	TM-Bacteria	0.000	0.000	0.000
Clustering.coefficient	TM-Bacteria	1.000	0.005	0.001
No.clusters	TM-Bacteria	226.000	19.050	4.036
Centralization.degree	TM-Bacteria	0.006	0.009	0.001
Centralization.betweenness	TM-Bacteria	0.000	0.026	0.005
Centralization.closeness	TM-Bacteria	0.000	0.006	0.002
Num.edges	CW-Fungi	4815.000	4815.000	0.000
Num.pos.edges	CW-Fungi	4386.000	0.000	0.000
Num.neg.edges	CW-Fungi	429.000	0.000	0.000
Num.vertices	CW-Fungi	400.000	400.000	0.000
Connectance	CW-Fungi	0.060	0.060	0.000
Average.degree	CW-Fungi	24.075	24.075	0.000
Average.path.length	CW-Fungi	2.805	2.159	0.001
Diameter	CW-Fungi	5.042	3.000	0.000
Edge.connectivity	CW-Fungi	0.000	11.300	1.611
Clustering.coefficient	CW-Fungi	0.933	0.060	0.001
No.clusters	CW-Fungi	7.000	1.000	0.000
Centralization.degree	CW-Fungi	0.040	0.037	0.005
Centralization.betweenness	CW-Fungi	0.009	0.005	0.001
Centralization.closeness	CW-Fungi	0.002	0.084	0.007
Num.edges	TW-Fungi	7166.000	7166.000	0.000
Num.pos.edges	TW-Fungi	5857.000	0.000	0.000
Num.neg.edges	TW-Fungi	1309.000	0.000	0.000
Num.vertices	TW-Fungi	400.000	400.000	0.000
Connectance	TW-Fungi	0.090	0.090	0.000
Average.degree	TW-Fungi	35.830	35.830	0.000
Average.path.length	TW-Fungi	2.667	1.946	0.001
Diameter	TW-Fungi	5.975	3.000	0.000
Edge.connectivity	TW-Fungi	0.000	20.310	1.704
Clustering.coefficient	TW-Fungi	0.983	0.090	0.001
No.clusters	TW-Fungi	7.000	1.000	0.000
Centralization.degree	TW-Fungi	0.118	0.045	0.006
Centralization.betweenness	TW-Fungi	0.015	0.003	0.001
Centralization.closeness	TW-Fungi	0.002	0.041	0.004

Num.edges	CW-Bacteria	3728.000	3728.000	0.000
Num.pos.edges	CW-Bacteria	2846.000	0.000	0.000
Num.neg.edges	CW-Bacteria	882.000	0.000	0.000
Num.vertices	CW-Bacteria	912.000	912.000	0.000
Connectance	CW-Bacteria	0.009	0.009	0.000
Average.degree	CW-Bacteria	8.175	8.175	0.000
Average.path.length	CW-Bacteria	1.326	3.480	0.004
Diameter	CW-Bacteria	3.942	6.070	0.256
Edge.connectivity	CW-Bacteria	0.000	0.830	0.533
Clustering.coefficient	CW-Bacteria	0.913	0.009	0.001
No.clusters	CW-Bacteria	151.000	1.250	0.458
Centralization.degree	CW-Bacteria	0.028	0.011	0.001
Centralization.betweenness	CW-Bacteria	0.000	0.009	0.002
Centralization.closeness	CW-Bacteria	0.000	0.075	0.017
Num.edges	TW-Bacteria	1689.000	1689.000	0.000
Num.pos.edges	TW-Bacteria	999.000	0.000	0.000
Num.neg.edges	TW-Bacteria	690.000	0.000	0.000
Num.vertices	TW-Bacteria	892.000	892.000	0.000
Connectance	TW-Bacteria	0.004	0.004	0.000
Average.degree	TW-Bacteria	3.787	3.787	0.000
Average.path.length	TW-Bacteria	1.000	5.194	0.029
Diameter	TW-Bacteria	1.000	11.160	0.788
Edge.connectivity	TW-Bacteria	0.000	0.000	0.000
Clustering.coefficient	TW-Bacteria	1.000	0.004	0.001
No.clusters	TW-Bacteria	240.000	21.750	3.996
Centralization.degree	TW-Bacteria	0.011	0.009	0.001
Centralization.betweenness	TW-Bacteria	0.000	0.028	0.006
Centralization.closeness	TW-Bacteria	0.000	0.005	0.001

Supplementary Table 6: The Top 10 relative abundance of microbes in the control and coated samples.

Group	Genus	Relative abundance
CM	Aspergillus	0.010
CM	Curvularia	0.014
CM	Fusarium	0.004
CM	Humicola	0.027
CM	Mortierella	0.007
CM	Penicillium	0.009
CM	Rhizoctonia	0.006
CM	Talaromyces	0.108
CM	Trichoderma	0.017
CM	Waitea	0.011
TM	Aspergillus	0.008
TM	Curvularia	0.016
TM	Fusarium	0.045
TM	Humicola	0.046
TM	Mortierella	0.009
TM	Penicillium	0.001
TM	Rhizoctonia	0.001
TM	Talaromyces	0.002
TM	Trichoderma	0.068
TM	Waitea	0.000
CW	Ascobolus	0.007
CW	Aspergillus	0.010
CW	Conocybe	0.014
CW	Curvularia	0.021
CW	Humicola	0.069
CW	Mortierella	0.010
CW	Penicillium	0.054
CW	Sonoraphlyctis	0.012
CW	Talaromyces	0.003
CW	Trichoderma	0.012
TW	Ascobolus	0.005
TW	Aspergillus	0.013
TW	Conocybe	0.079
TW	Curvularia	0.016
TW	Humicola	0.044
TW	Mortierella	0.009
TW	Penicillium	0.063
TW	Sonoraphlyctis	0.001
TW	Talaromyces	0.056
TW	Trichoderma	0.033

Supplementary Table 7: The topological properties of nodes in the module.

	Nodes	Igraph.degree	Igraph.closeness	Igraph.betweenness	Igraph.cen.degree
CW	ASV_257	2	0.095	26.000	2
CW	ASV_7	3	0.105	93.000	3
CW	ASV_69	2	0.099	31.667	2
CW	ASV_130	2	0.105	25.000	2
CW	ASV_68	3	0.114	142.500	3
CW	ASV_177	3	0.107	30.500	3
CW	ASV_13	3	0.107	30.500	3
CW	ASV_9	1	0.030	0.000	1
CW	ASV_97	3	0.101	58.000	3
CW	ASV_23	3	0.104	36.333	3
CW	ASV_719	3	0.105	38.833	3
CW	ASV_31	2	0.106	26.000	2
CW	ASV_633	3	0.112	119.000	3
CW	ASV_3	3	0.091	26.000	3
CW	ASV_744	2	0.104	21.500	2
CW	ASV_1004	3	0.110	123.000	3
CW	ASV_146	3	0.104	59.000	3
CW	ASV_381	3	0.097	24.000	3
CW	ASV_176	3	0.097	24.000	3
CW	ASV_361	1	0.088	0.000	1
CW	ASV_344	1	0.030	0.000	1
CW	ASV_247	1	0.097	0.000	1
CW	ASV_14	2	0.098	30.667	2
CW	ASV_139	1	0.030	0.000	1
CW	ASV_22	3	0.102	79.000	3
CW	ASV_8	4	0.111	101.500	4
CW	ASV_78	1	0.030	0.000	1
CW	ASV_155	1	0.093	0.000	1
CW	ASV_187	1	0.096	0.000	1
CW	ASV_62	2	0.109	94.000	2
CW	ASV_6	1	0.085	0.000	1
CW	ASV_185	3	0.104	92.000	3
CW	ASV_271	1	0.030	0.000	1
CW	ASV_121	1	0.030	0.000	1
TW	ASV_1004	4	0.072	92.000	4
TW	ASV_116	4	0.067	4.133	4
TW	ASV_13	3	0.066	0.000	3
TW	ASV_130	1	0.026	0.000	1
TW	ASV_139	1	0.027	0.000	1
TW	ASV_14	4	0.029	1.000	4
TW	ASV_146	1	0.070	0.000	1
TW	ASV_154	4	0.029	0.333	4

TW	ASV_155	3	0.065	26.000	3
TW	ASV_176	2	0.065	26.000	2
TW	ASV_177	1	0.027	0.000	1
TW	ASV_185	4	0.069	24.000	4
TW	ASV_187	2	0.029	0.000	2
TW	ASV_22	5	0.070	68.167	5
TW	ASV_23	4	0.068	24.000	4
TW	ASV_247	4	0.072	66.333	4
TW	ASV_257	1	0.070	0.000	1
TW	ASV_263	4	0.069	22.000	4
TW	ASV_271	4	0.067	4.133	4
TW	ASV_3	3	0.071	162.500	3
TW	ASV_31	3	0.069	74.100	3
TW	ASV_344	4	0.065	3.000	4
TW	ASV_361	5	0.074	199.167	5
TW	ASV_381	4	0.069	24.000	4
TW	ASV_6	3	0.073	191.000	3
TW	ASV_62	2	0.072	170.000	2
TW	ASV_633	4	0.068	24.000	4
TW	ASV_67	2	0.027	1.000	2
TW	ASV_68	5	0.029	2.333	5
TW	ASV_69	1	0.062	0.000	1
TW	ASV_7	4	0.067	54.300	4
TW	ASV_719	4	0.029	0.333	4
TW	ASV_744	3	0.066	0.000	3
TW	ASV_78	4	0.069	58.900	4
TW	ASV_8	4	0.072	58.333	4
TW	ASV_828	3	0.029	0.000	3
TW	ASV_9	1	0.026	0.000	1
TW	ASV_942	1	0.062	0.000	1
TW	ASV_97	6	0.068	16.933	6
CM	ASV_244	2	0.048	0.000	2
CM	ASV_132	2	0.048	0.000	2
CM	ASV_187	4	0.055	0.333	4
CM	ASV_6	3	0.055	0.000	3
CM	ASV_176	3	0.055	0.000	3
CM	ASV_579	5	0.056	1.167	5
CM	ASV_619	4	0.055	0.333	4
CM	ASV_366	4	0.053	0.000	4
CM	ASV_563	4	0.053	0.000	4
CM	ASV_698	4	0.053	0.000	4
CM	ASV_303	4	0.053	0.000	4
CM	ASV_361	1	0.045	0.000	1
CM	ASV_68	1	0.048	0.000	1

CM	ASV_174	1	0.048	0.000	1
CM	ASV_258	1	0.045	0.000	1
CM	ASV_817	1	0.045	0.000	1
CM	ASV_198	2	0.048	0.000	2
CM	ASV_78	5	0.056	1.167	5
CM	ASV_199	4	0.053	0.000	4
CM	ASV_116	1	0.045	0.000	1
CM	ASV_798	2	0.048	1.000	2
CM	ASV_744	1	0.045	0.000	1
CM	ASV_240	1	0.045	0.000	1
TM	ASV_155	6	0.308	41.361	6
TM	ASV_798	4	0.291	41.058	4
TM	ASV_293	7	0.373	44.363	7
TM	ASV_361	6	0.353	59.833	6
TM	ASV_698	7	0.373	44.363	7
TM	ASV_199	11	0.456	99.348	11
TM	ASV_68	6	0.363	0.000	6
TM	ASV_377	12	0.432	70.888	12
TM	ASV_257	8	0.357	20.838	8
TM	ASV_366	10	0.410	23.031	10
TM	ASV_190	6	0.357	76.202	6
TM	ASV_668	10	0.410	23.031	10
TM	ASV_258	9	0.390	95.952	9
TM	ASV_817	11	0.436	61.449	11
TM	ASV_653	11	0.423	61.724	11
TM	ASV_600	3	0.333	1.751	3
TM	ASV_381	7	0.373	12.235	7
TM	ASV_244	8	0.427	153.850	8
TM	ASV_579	9	0.427	87.816	9
TM	ASV_334	4	0.333	2.693	4
TM	ASV_371	4	0.311	1.333	4
TM	ASV_744	7	0.436	102.002	7
TM	ASV_185	5	0.410	42.198	5
TM	ASV_303	10	0.456	101.769	10
TM	ASV_145	6	0.353	15.504	6
TM	ASV_174	1	0.237	0.000	1
TM	ASV_176	2	0.277	20.762	2
TM	ASV_116	4	0.380	6.971	4
TM	ASV_466	3	0.275	8.812	3
TM	ASV_240	3	0.275	8.812	3
TM	ASV_215	2	0.295	30.711	2
TM	ASV_132	4	0.325	11.549	4
TM	ASV_633	3	0.299	53.480	3
TM	ASV_209	3	0.304	4.183	3

TM	ASV_619	2	0.304	0.000	2
TM	ASV_22	5	0.315	28.651	5
TM	ASV_198	10	0.410	23.031	10
TM	ASV_78	7	0.398	45.884	7
TM	ASV_187	4	0.342	47.344	4
TM	ASV_410	7	0.373	32.906	7
TM	ASV_6	2	0.263	13.271	2
TM	ASV_563	3	0.306	1.038	3

Supplementary Table 8: List of preserved module pairs, the number of overlapping and non-overlapping nodes in paired modules.

Module1	Module2	Both	Nodes only in module1	Nodes only in module2	Nodes absent from both modules	Fisher exact test p value	Adjusted p value**
TM_M7	TM-TRI-remove_M39	0	42	51	707	1.00	1.00
TM_M7	TM-TRI-remove_M40	3	39	38	723	0.36	1.00
TM_M7	TM-TRI-remove_M41	5	37	36	727	0.06	1.00
TM_M7	TM-TRI-remove_M42	5	37	25	738	0.02	0.47
TM_M7	TM-TRI-remove_M10	4	38	21	741	0.04	1.00
TM_M7	TM-TRI-remove_M43	1	41	20	739	0.68	1.00
TM_M7	TM-TRI-remove_M32	2	40	15	745	0.22	1.00
TM_M7	TM-TRI-remove_M44	1	41	13	746	0.53	1.00
TM_M7	TM-TRI-remove_M52	0	42	9	749	1.00	1.00
TM_M7	TM-TRI-remove_M18	2	37	5	758	0.06	1.00
TM_M7	TM-TRI-remove_M19	0	42	9	749	1.00	1.00
TM_M7	TM-TRI-remove_M14	1	41	8	751	0.39	1.00
TM_M7	TM-TRI-remove_M53	0	42	7	751	1.00	1.00
TM_M7	TM-TRI-remove_M59	1	41	8	751	0.39	1.00
TM_M7	TM-TRI-remove_M4	0	42	9	749	1.00	1.00
TM_M7	TM-TRI-remove_M37	0	42	7	751	1.00	1.00
TM_M7	TM-TRI-remove_M55	0	42	6	752	1.00	1.00
TM_M7	TM-TRI-remove_M54	1	41	5	754	0.28	1.00
TM_M7	TM-TRI-remove_M29	0	42	6	752	1.00	1.00
TM_M7	TM-TRI-remove_M20	0	42	8	750	1.00	1.00
TM_M7	TM-TRI-remove_M57	0	42	5	753	1.00	1.00
TM_M7	TM-TRI-remove_M22	0	42	7	751	1.00	1.00
TM_M7	TM-TRI-remove_M56	0	42	5	753	1.00	1.00
TM_M7	TM-TRI-remove_M24	1	41	4	755	0.24	1.00
TM_M7	TM-TRI-remove_M23	0	42	5	753	1.00	1.00
TM_M7	TM-TRI-remove_M3	0	42	5	753	1.00	1.00
TM_M7	TM-TRI-remove_M1	0	42	6	752	1.00	1.00
TM_M7	TM-TRI-remove_M17	0	42	21	737	1.00	1.00
TM_M7	TM-TRI-remove_M11	0	42	5	753	1.00	1.00
TW_M3	TW-TRI-remove_M15	0	41	119	640	1.00	1.00
TW_M3	TW-TRI-remove_M7	1	40	48	712	0.93	1.00
TW_M3	TW-TRI-remove_M17	0	41	42	717	1.00	1.00
TW_M3	TW-TRI-remove_M18	0	41	37	722	1.00	1.00
TW_M3	TW-TRI-remove_M19	0	41	20	739	1.00	1.00
TW_M3	TW-TRI-remove_M20	2	39	15	746	0.21	1.00
TW_M3	TW-TRI-remove_M21	2	39	12	749	0.16	1.00
TW_M3	TW-TRI-remove_M8	0	41	14	745	1.00	1.00
TW_M3	TW-TRI-remove_M16	3	38	15	747	0.06	0.95
TW_M3	TW-TRI-remove_M14	4	37	11	752	0.01	0.08

TW_M3	TW-TRI-remove_M22	2	39	4	757	0.03	0.54
TW_M3	TW-TRI-remove_M13	1	40	7	753	0.34	1.00
TW_M3	TW-TRI-remove_M11	2	39	4	757	0.03	0.54
TW_M3	TW-TRI-remove_M1	2	39	12	749	0.16	1.00
TW_M3	TW-TRI-remove_M9	0	41	5	754	1.00	1.00
TM_M7	TM-TRI-reduce_M47	0	42	67	651	1.00	1.00
TM_M7	TM-TRI-reduce_M48	0	42	63	655	1.00	1.00
TM_M7	TM-TRI-reduce_M35	0	42	54	664	1.00	1.00
TM_M7	TM-TRI-reduce_M15	0	42	40	678	1.00	1.00
TM_M7	TM-TRI-reduce_M38	0	42	33	685	1.00	1.00
TM_M7	TM-TRI-reduce_M32	0	42	25	693	1.00	1.00
TM_M7	TM-TRI-reduce_M50	0	42	22	696	1.00	1.00
TM_M7	TM-TRI-reduce_M52	0	42	16	702	1.00	1.00
TM_M7	TM-TRI-reduce_M55	2	40	6	714	1.00	1.00
TM_M7	TM-TRI-reduce_M54	2	40	6	714	1.00	1.00
TM_M7	TM-TRI-reduce_M56	0	42	7	711	1.00	1.00
TM_M7	TM-TRI-reduce_M13	1	41	7	712	1.00	1.00
TM_M7	TM-TRI-reduce_M57	0	42	6	712	1.00	1.00
TM_M7	TM-TRI-reduce_M33	0	42	7	711	1.00	1.00
TM_M7	TM-TRI-reduce_M17	4	38	5	717	1.00	1.00
TM_M7	TM-TRI-reduce_M43	0	42	7	711	1.00	1.00
TM_M7	TM-TRI-reduce_M30	0	42	6	712	1.00	1.00
TM_M7	TM-TRI-reduce_M59	0	42	5	713	1.00	1.00
TM_M7	TM-TRI-reduce_M45	0	42	6	712	1.00	1.00
TM_M7	TM-TRI-reduce_M18	0	42	6	712	1.00	1.00
TM_M7	TM-TRI-reduce_M58	1	41	4	715	1.00	1.00
TM_M7	TM-TRI-reduce_M12	1	41	5	714	1.00	1.00
TM_M7	TM-TRI-reduce_M2	0	42	5	713	1.00	1.00
TM_M7	TM-TRI-reduce_M31	0	42	5	713	1.00	1.00
TM_M7	TM-TRI-reduce_M23	1	41	4	715	1.00	1.00
TM_M7	TM-TRI-reduce_M40	1	41	23	696	1.00	1.00
TM_M7	TM-TRI-reduce_M36	0	42	5	713	1.00	1.00
TM_M7	TM-TRI-reduce_M5	0	42	5	713	1.00	1.00
TM_M7	TM-TRI-reduce_M20	0	42	5	713	1.00	1.00
TM_M7	TM-TRI-reduce_M8	5	37	16	707	1.00	1.00
TW_M3	TW-TRI-reduce_M18	0	41	121	638	1.00	1.00
TW_M3	TW-TRI-reduce_M1	1	40	51	709	0.94	1.00
TW_M3	TW-TRI-reduce_M19	1	40	44	716	0.91	1.00
TW_M3	TW-TRI-reduce_M20	2	39	39	722	0.63	1.00
TW_M3	TW-TRI-reduce_M17	2	39	23	738	0.37	1.00
TW_M3	TW-TRI-reduce_M29	0	41	16	743	1.00	1.00
TW_M3	TW-TRI-reduce_M30	2	39	9	752	0.10	1.00
TW_M3	TW-TRI-reduce_M12	1	40	11	749	0.47	1.00
TW_M3	TW-TRI-reduce_M15	2	39	10	751	0.12	1.00

TW_M3	TW-TRI-reduce_M14	2	39	9	752	0.10	1.00
TW_M3	TW-TRI-reduce_M9	0	41	8	751	1.00	1.00
TW_M3	TW-TRI-reduce_M4	1	40	7	753	0.34	1.00
TW_M3	TW-TRI-reduce_M21	3	38	6	756	0.01	0.32
TW_M3	TW-TRI-reduce_M31	2	39	4	757	0.03	1.00
TW_M3	TW-TRI-reduce_M10	0	41	9	750	1.00	1.00
TW_M3	TW-TRI-reduce_M8	4	37	22	741	0.04	1.00
TW_M3	TW-TRI-reduce_M6	2	39	8	753	0.09	1.00
TW_M3	TW-TRI-reduce_M33	2	39	3	758	0.02	0.88
TW_M3	TW-TRI-reduce_M7	1	40	8	752	0.38	1.00
TW_M3	TW-TRI-reduce_M22	2	39	8	753	0.09	1.00
TW_M3	TW-TRI-reduce_M32	1	40	4	756	0.23	1.00
TW_M3	TW-TRI-reduce_M5	2	39	8	753	0.09	1.00
TW_M3	TW-TRI-reduce_M16	2	39	9	752	0.10	1.00
TW_M3	TW-TRI-reduce_M11	2	39	6	755	0.06	1.00
TW_M3	TW-TRI-reduce_M24	1	40	5	755	0.27	1.00
TW_M3	TW-TRI-reduce_M23	1	40	4	756	0.23	1.00
TW_M3	TW-TRI-reduce_M36	2	39	5	756	0.05	1.00
TW_M3	TW-TRI-reduce_M34	0	41	6	753	1.00	1.00
TW_M3	TW-TRI-reduce_M2	1	40	5	755	0.27	1.00
TW_M3	TW-TRI-reduce_M3	5	36	6	758	0.00	0.00
TW_M3	TW-TRI-reduce_M40	2	39	21	740	0.33	1.00
TW_M3	TW-TRI-reduce_M25	1	40	4	756	0.23	1.00
TW_M3	TW-TRI-reduce_M26	0	41	6	753	1.00	1.00
TW_M3	TW-TRI-reduce_M28	1	40	4	756	0.23	1.00
TW_M3	TW-TRI-reduce_M27	0	41	6	753	1.00	1.00
TW_M3	TW-TRI-reduce_M13	1	40	4	756	0.23	1.00
TW_M3	TW-TRI-reduce_M39	1	40	5	755	0.27	1.00
TW_M3	TW-TRI-reduce_M37	1	40	5	755	0.27	1.00
